# Early Diagnosis and Intervention in Cystic Fibrosis: Imagining the Unimaginable

**DOI:** 10.3389/fped.2020.608821

**Published:** 2021-01-11

**Authors:** Andrea M. Coverstone, Thomas W. Ferkol

**Affiliations:** ^1^Department of Pediatrics, Washington University School of Medicine, St. Louis, MO, United States; ^2^Department of Cell Biology and Physiology, Washington University School of Medicine, St. Louis, MO, United States

**Keywords:** cystic fibrosis, cystic fibrosis transmembrane conductance regulator, corrector, potentiator, immunoreactive trypsin(ogen), sweat chloride test, newborn screening

## Abstract

Cystic fibrosis is the most common life-shortening genetic disease affecting Caucasians, clinically manifested by fat malabsorption, poor growth and nutrition, and recurrent sinopulmonary infections. Newborn screening programs for cystic fibrosis are now implemented throughout the United States and in many nations worldwide. Early diagnosis and interventions have led to improved clinical outcomes for people with cystic fibrosis. Newer cystic fibrosis transmembrane conductance regulator potentiators and correctors with mutation-specific effects have increasingly been used in children, and these agents are revolutionizing care. Indeed, it is possible that highly effective modulator therapy used early in life could profoundly affect the trajectory of cystic fibrosis lung disease, and primary prevention may be achievable.

## Introduction

Newborn screening (NBS) programs were first established almost 60 years ago in the United States after the seminal discovery that phenylalanine could be detected from a dried blood spot, ultimately leading to early diagnosis of phenylketonuria and avoidance of the severe neurocognitive complications characteristic of this inherited metabolic disorder (1). Other screening programs emerged, generally adhering to basic principles outlined in a report commissioned by the World Health Organization (2), and the number of diseases tested has grown in the last several decades. While there is variability between programs, some states and countries screen for as many as 50 treatable metabolic conditions, endocrinopathies, hemoglobinopathies, and genetic diseases, like cystic fibrosis (CF).

Occurring in roughly 1 in 3,000 live births in the United States, based on epidemiological and neonatal screening data, CF is the most common, life-shortening inherited disease of Caucasians (3). CF is caused by defective CF transmembrane conductance regulator (CFTR), a cAMP-regulated anion transporter expressed on the surface of various epithelia. Functionally linked to the epithelial sodium channel and other apical channels, CFTR abnormalities lead to reduced epithelial chloride conductance and sodium absorption, resulting in dehydration of the periciliary fluid layer and mucus on the airway surface that impairs mucociliary clearance (4, 5). Innate defenses are also compromised by altered bicarbonate secretion in the CF airway (6, 7). Together, these changes lead to progressive airway obstruction, allowing bacterial infection to become established and provoking a persistent neutrophilic inflammatory response that results in the gradual destruction of the airways and ultimately respiratory failure.

Before implementation of NBS for CF, children were typically diagnosed after developing symptoms consistent with fat malabsorption, often leading to nutritional failure. Some were identified shortly after birth when they presented with meconium ileus, which occurs in roughly 15% of children with CF (8, 9). Recurrent respiratory infections, often misdiagnosed as asthma or bronchiolitis, would occur during infancy, but are more common in older children. Indeed, children with milder, pancreatic sufficient phenotypes are often recognized later as their respiratory symptoms become more prevalent.

Adoption of NBS in many nations has led to earlier diagnosis and treatment, and the life expectancy of a child born with CF in many parts of the world has steadily improved (10, 11). In addition, newer, small molecule therapeutics have begun to dramatically change the disease (12, 13). In this article, we will review NBS for CF and describe existing and emerging therapies that have impacted the progressive respiratory decline of people with CF and how they may avert lung disease and other complications even before they begin.

## NBS For CF

Early attempts to screen neonates for CF over 40 years ago relied on measuring albumin content in dried meconium (14), which had a high false-positive rate. However, it was discovered that young infants with CF had elevated, circulating levels of pancreatic enzymes and proenzymes, even children with pancreatic sufficient forms of the disease. In particular, trypsinogen, a pancreatic enzyme precursor released from the inflamed exocrine pancreas caused by inspissated secretions and destruction of acinar cells, can be detected in the blood of neonates with CF. Indeed, over 40 years ago, Crossley and colleagues showed that the serum immunoreactive trypsinogen (IRT) could be measured in blood spots dried on the Guthrie cards (15), and the ability to measure this analyte was paramount for development of a broad, population-based newborn program.

In the United States, methods for NBS differ between states and countries, but all invariably use some form of the IRT measurements as part of the screening process. It is important to note that IRT concentrations can be elevated in the absence of CF, particularly in neonates who are premature, have low Apgar scores, or experience perinatal stress. It was recognized that a single-tier approach had a lower sensitivity, so in the United States, most states have adopted two-tier protocols that involve serial IRT measurements repeated 1 to 2 weeks apart if the initial value is elevated, or IRT followed by genetic testing for specific *CFTR* mutations if abnormal. Some states have adopted a third-tier, using a protocol that involves repeated measurements of IRT levels, with DNA analysis performed if both concentrations are above the designated threshold (16, 17). Uniquely, California, a state that has a racially diverse population, has incorporated *CFTR* sequencing in their screen to identify CF in neonates with high IRT concentrations and only one mutation in their genetic panel (18).

The threshold defining an elevated IRT level varies between states. While some states will apply an absolute concentration to prompt further testing, others use percentile cutoffs that improve specificity. Serial IRT approaches without genetic analysis has benefits, allowing for identification of individuals with less common *CFTR* mutations in certain populations (19), but delaying time to a positive screen because the second specimen is obtained later. Genetic panels used in NBS are variable, and the number of *CFTR* mutations analyzed may differ from state-to-state. Genetic testing has the advantage of identifying people who are heterozygous for *CFTR* mutations, but also more likely to identify patients with mutations but normal or equivocal sweat chloride levels, referred to as CFTR-related metabolic syndrome or, in Europe, CF screen-positive, inconclusive diagnosis.

## Adoption and Evolution of CF NBS

In the United States, NBS for CF was slowly accepted, given the relative absence of data showing benefits of early diagnosis. Indeed, even the Cystic Fibrosis Foundation was hesitant to make a recommendation regarding NBS, stating the benefits of presymptomatic and early treatment were controversial (20). Nevertheless, screening programs were established in North America. In 1982, Colorado became the first state to implement NBS for CF, followed shortly thereafter by Wisconsin. Initially developed as part of a decade-long randomized controlled trial (21), NBS was added to the Wisconsin state-wide program in 1994 (22). A workshop held by the Centers for Disease Control and Prevention and Cystic Fibrosis Foundation in 2003 evaluated diagnostic testing and decision-making and provided recommendations for best practices for screening for CF (23). At the time of its publication, only eight states had implemented an NBS program, but within 7 years, all 50 states and Washington, DC, had screening programs for CF, with Texas being the last state to implement screening.

Similar to the US experience, there is considerable variability in screening programs among nations. Certainly, disease prevalence plays an influential role in the need for screening and early diagnosis (24, 25). Worldwide, Australia and New Zealand were pioneers, establishing NBS programs in 1981 (26). During the past two decades, the number of programs has rapidly increased in Europe (25, 27), with more than 20 European countries performing NBS at some level. Indeed, alternative screening approaches have been adopted in some countries. For instance, a four-tier screening algorithm was created in the Netherlands that involves measurement of IRT and pancreatitis-associated protein levels from the same dried blood spot (28), *CFTR* mutation panel, and, if indicated, extended genomic analysis. The Dutch experience highlights the complexity of such programs, and a reminder NBS is exactly that, a screening tool and not a diagnostic test.

Often overlooked, successful NBS depends on the accuracy of diagnostic testing. The diagnosis of CF is based on elevated sweat chloride concentrations (29). Any clinical concerns for CF, regardless of the screening result, are an indication for sweat chloride measurement. While other approaches are available, the only reliable, validated diagnostic test for measuring sweat chloride concentration is the quantitative pilocarpine iontophoresis test, performed according to Clinical and Laboratory Standards Institute guidelines (30).

## Benefits of NBS For CF

NBS, regardless of disease, is successful only if early identification is feasible using simple, cost-effective means and can lead to improved clinical outcomes. For decades, the diagnosis of CF required clinical suspicion. Before screening, the median age of diagnosis was 6 months in the United States, and nearly 70% of affected children were identified by their first birthday (31). Malnutrition occurs early in life (32), and pulmonary involvement can begin early in infancy, despite the child appearing asymptomatic (33, 34). With widespread adoption of NBS in the United States, the age at diagnosis has shifted to <1 month, often before the child is symptomatic.

There was early evidence from the Netherlands almost 5 decades ago that screening in the neonatal period was associated with a survival benefit (35). While CF was added relatively late to US programs, there was growing evidence that delayed diagnosis would have serious implications for affected people. Because of low mortality rates in children, it is difficult to establish survival differences between screened and unscreened children with CF. Using data from the US Cystic Fibrosis Foundation Patient Registry of more than 27,000 patients, children identified by screening within a month of age and treated early had better survival compared to counterparts diagnosed symptomatically (36), supported by several subsequent studies (37–39).

The best evidence clearly showing benefits of NBS is its effect on early nutrition and growth. Before screening, children with CF were often diagnosed after becoming severely malnourished with vitamin deficiencies. These children frequently failed to achieve their growth potential and had evidence of impaired development of cognitive function, likely related to malnutrition. Investigators in Wisconsin unsurprisingly found that the diagnosis of CF was confirmed by a positive sweat test at a much younger age in a screened cohort as compared to controls. Moreover, children with CF identified by screening had significantly better height, weight, and head circumference percentiles, and these differences persisted throughout infancy and early childhood, especially the children who had pancreatic insufficiency and homozygous for the *Phe508del* mutation (40). Recently, a multicenter, longitudinal, observational cohort study examined the nutritional health of 231 American children with CF identified by NBS over a 3-year period and found significant improvement in nutritional status, with normalization of weight in the first year of life (41).

Malnourished children with CF have increased risk of chronic lung disease. A large study of 931 children with CF examining the effect of early nutrition on the development of lung disease highlighted the importance of earlier intervention. Children with better nutritional indices at 3 years of age had higher lung function measures at the age of 6 years (42). Thus, early diagnosis in order to optimize the nutritional status by starting enzymes at diagnosis and adding nutritional supplements as indicated can lead to improved pulmonary health.

There have been several studies that show children diagnosed with CF following NBS have fewer complications than those who were symptomatic at the time of diagnosis. Australian investigators compared the outcomes of children with CF identified early from NBS or diagnosed late. Unscreened patients had reduced height, lower pulmonary function measures, and increased rates of infection and colonization with *Pseudomonas aeruginosa* (43). Respiratory benefits persisted into adolescence (44, 45). Others have similarly shown benefits in the US and UK populations, with reduced *P. aeruginosa* infections, reduced treatment burden, and fewer hospitalizations (46–48). These findings emphasize not only the importance of early diagnosis and detection, but also the need for continued improvement of screening protocols with genetic advances.

In addition to the clinical benefits from early identification, screening programs for CF have economic benefits, with several studies revealing its cost-effectiveness (49, 50). Additionally, investigators have reported that the incidence or CFTR allele distribution decreased following implementation of NBS, although these observations need to be confirmed in larger studies (51, 52).

## Implications of NBS In The ERA of Modulator Therapies

Management of CF has been directed at the downstream consequences of CFTR dysfunction, incorporating antibiotics, anti-inflammatory agents, inhaled mucolytics, and airway clearance techniques. Pancreatic enzyme replacement therapy and vitamin supplements treat pancreatic insufficiency and prevent nutritional deficits. These treatments have led to longer lives, even before widespread implementation of NBS. However, the emergence of novel small molecule therapeutics that target the basic defects has raised hope that CF lung disease can be prevented before it starts. These newer CFTR potentiators and correctors have mutation-specific effects that can restore CFTR function. These agents are revolutionizing care and have reduced respiratory symptoms, exacerbation frequency, and slowed progression of lung disease in people with CF (12, 13).

When ivacaftor was approved by the US Food and Drug Administration (FDA) 8 years ago, preceding clinical trials showed dramatic improvements in sweat chloride values along with improvements in weight gain, pulmonary exacerbation rates, and lung function measures in patients with *G551D* mutations, a class 3 *CFTR* gating defect (12, 53). Subsequent studies revealed improved lung mucociliary clearance that was sustained 3 months following treatment, which correlated with forced expiratory volume in 1 s (FEV_1_) (54). More recently, several studies then evaluated the effectiveness of ivacaftor in younger children (55–57). A phase 3, multicenter trial examined the ivacaftor pharmacokinetics in young children and its effect on sweat chloride concentrations, growth parameters, and markers of exocrine pancreatic function. Growth measures for age were normal throughout the study, and pancreatic function biomarkers also improved, suggesting that ivacaftor preserved exocrine pancreatic function (56). Other studies have shown beneficial nutritional effects in preschool children (55, 58). These findings were surprising. In CF, the exocrine pancreas is involved before birth, with obstruction of small ducts and acini seen as early as the second trimester (59). Disease progresses after birth, with pancreatic inflammation, fibrosis, and fatty infiltration, once thought to occur in early infancy (60).

In secondary analyses of GOAL (*G551D* Observational Study), lung clearance indices were significantly improved in treated children within 1 month of starting treatment and were maintained 6 months after beginning therapy (61). In fact, ivacaftor is now approved for use in children with certain mutations as young as 4 months of age.

Since the development of ivacaftor (Kalydeco (R)), three other therapies have been approved for use in the United States (62). Lumacaftor–ivacaftor (Orkambi®) was initially approved for use in 2015 for those with homozygous *Phe508del* mutations and is now available for use down to 2 years of age. Tezacaftor–ivacaftor (Symdeko®) was approved in 2018 and may now be used for those 6 years or older who have homozygous *Phe508del* mutations or *Phe508del* and a second specific mutation. Most recently, highly effective triple-drug therapy, elexacaftor–tezacaftor–ivacaftor (Trikafta® or Kaftrio®), was approved by the FDA and more recently the European Commission for use in CF patients 12 years or older who have one or two *Phe508del* mutations, which accounts for 90% of all affected individuals (13, 63). This treatment has shown the most promise in altering the clinical trajectory of those with CF. Trials showed marked reductions of sweat chloride and improved percent predicted FEV_1_ values (13).

Modulator therapy is increasingly used in younger children and even infants, raising the prospect that CF could be prevented before it begins (55, 56). However, to be successful, primary prevention requires early diagnosis and treatment. NBS is an essential part of the success of early diagnosis and with the advent of modulator therapy use in younger and younger children, critically important.

Given these improvements, although purely speculative, primary prevention may be achievable. Using genetically modified ferrets that harbor *CFTR G551D* mutations, investigators showed the potential benefits of *CFTR* modulators (64). Like other animal models for CF, the newborn ferret is prone to meconium ileus, with 80% experiencing severe intestinal obstruction that leads to early death. However, when pregnant jills were treated with ivacaftor (VX-770) late in pregnancy, kits homozygous for the *G551D* mutation had markedly reduced incidence of neonatal bowel obstruction.

**Figure 1:**
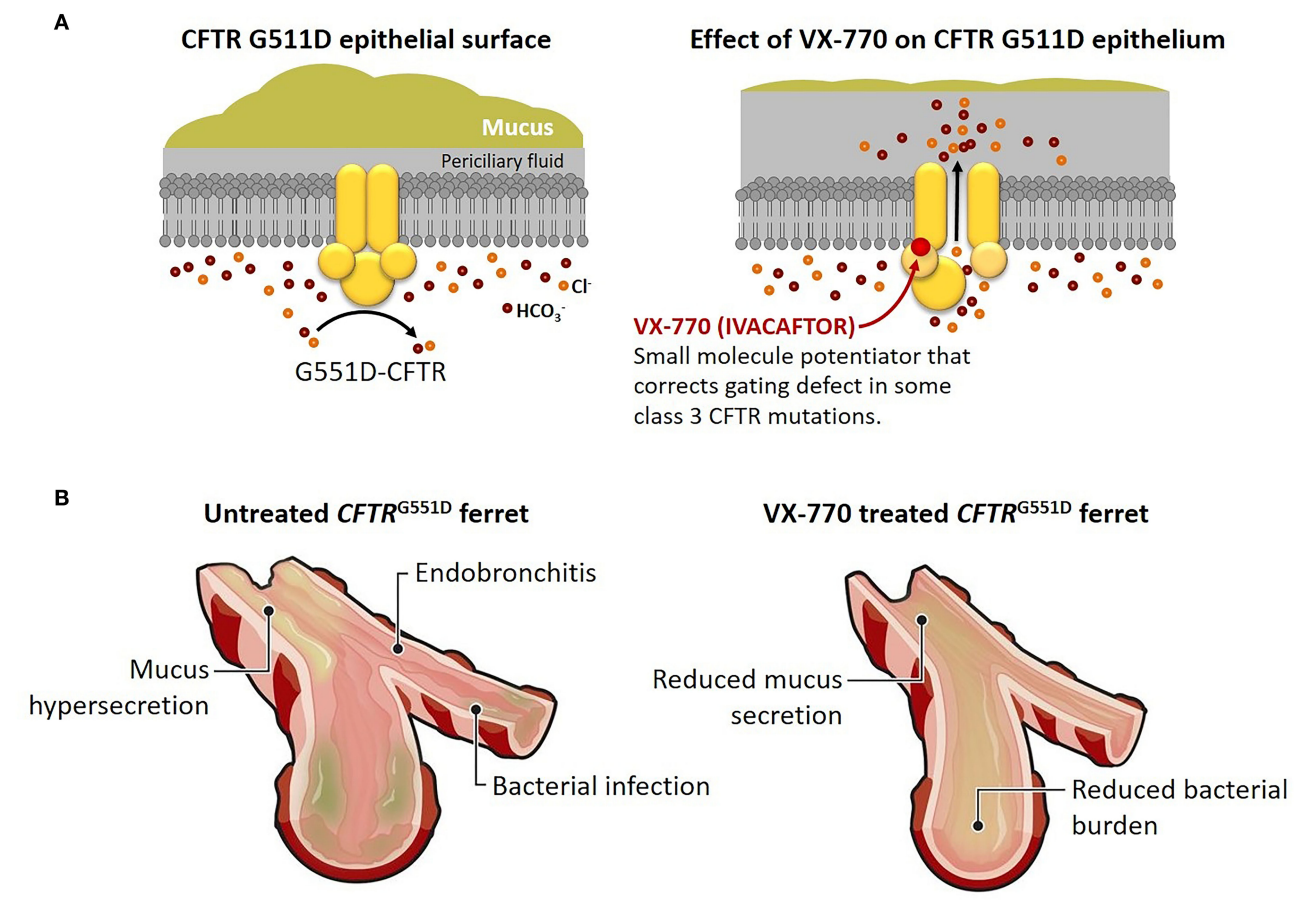
Early treatment with VX-770 prevents pathological changes in a cystic fibrosis animal model. **(A)** Effect of the CFTR potentiator VX-770 (ivacaftor) on ion channel gating of the CFTR G551D mutation. The *G551D* mutation abolishes ATP-dependent gating, which results in reduced channel open probability, but treatment with VX-770 alters activity of the mutant CFTR, leading to greater chloride ion and bicarbonate ion secretion, reduced sodium ion absorption, and hydration of epithelial surfaces. **(B)** Effect of prenatal and postnatal treatment with VX-770 (ivacaftor) on the airways of young ferrets with G551D mutation. Mucus accumulation, bacterial infection, and endobronchitis develop early in untreated airways of young kits with *G551D* mutations, but treated animals avoided bronchial infection and inflammation until the drug was discontinued. Similar effects were seen in other affected organs, including the pancreas, intestines, and genitourinary tract. Modified from Ferkol (65).

Postnatally, ivacaftor was administered to the kits, and they maintained pancreatic sufficiency and grew as well as wild-type littermates. In compound heterozygous (*G551D/KO*) ferrets, most remained pancreatic insufficient, but many maintained normal growth. Similarly, ivacaftor treatment protected the airways from bacterial infection and inflammation (Figure 1). Once treatment was discontinued; however, the benefits disappeared, and CF kits developed characteristic pancreatic and pulmonary pathology. These findings suggest the importance of early and sustained modulator treatment in maintaining *CFTR* function (65), and these agents are not a cure.

While fertility was not assessed, the vas deferens and epididymis appeared pathologically normal in male kits homozygous for the *G551D* mutation, in contrast to compound heterozygous (*G551D/KO*) ferrets. Thus, one could speculate obstructive azoospermia or congenital bilateral absence of the vas deferens could be prevented in certain patients. The pathogenesis of the male genitourinary defects begins *in utero*, likely related to accumulation of obstructing, thickened secretions that leads to degeneration of the vas deferens. Indeed, male fetuses with CF, between 12- and 18-week gestation, have a normal vas deferens, demonstrating that the defect occurs later in embryonic development (66).

It would be premature to consider clinical trials testing the efficacy of ivacaftor in preventing CF in neonates who have *G551D*. First, there would be few eligible subjects. Few people with CF are homozygous for class 3 *CFTR* defects (53). Moreover, treating a fetus by treating an unaffected pregnant mother would pose ethical issues; pregnant women and their unborn children are often excluded from pharmaceutical trials. These therapies are not without risk, including liver dysfunction and cataract development, and would likely prohibit use in an unaffected woman.

That said, we may soon have evidence of whether primary prevention of CF is feasible. In contrast to their male counterparts who have obstructive azoospermia, women with CF are generally fertile, and with improvements in care, a growing proportion are having children. Many women with CF are being treated with the newer, highly effective triple combination therapy, elexacaftor–tezacaftor–ivacaftor (13, 63). To maintain the mother's pulmonary and nutritional health, they often continue treatment throughout pregnancy at many centers.

While partners of pregnant women with CF typically undergo prenatal testing for *CFTR* mutations, occasionally they are missed, and children are born with CF. If their unborn child has CF and *Phe508del* mutation(s), he/she would indirectly be treated with elexacaftor–tezacaftor–ivacaftor *in utero*, as these small molecules can cross the placental barrier, thus leading to several interesting questions. Would combination therapy in this child prevent progressive airway disease, maintain pancreatic sufficiency, or preserve male fertility, paralleling what was described in the ferret model (65)? How would one assess the latter in young infants who typically do not have respiratory symptoms (67), and what would we use to demonstrate a treatment effect in the lung (67)? For primary prevention strategies to succeed, sensitive outcome measures are needed to detect the earliest changes in lung disease in infants and young children.

Furthermore, would it be unethical to withdraw a drug that prevented disease once the infant is born, despite lack of regulatory approval for young infants? If so, in the absence of clinical trials, how would we determine optimal dosing in this population?

Finally, would CFTR correction interfere with NBS of children born to women with CF, resulting in a false-negative screen? Could CFTR correction attenuate pancreatic injury and result in a negative IRT level? We may need to rethink our screening and diagnostic approach for such children.

While there are many significant gaps in available diagnostics and treatments between countries (68), we have entered a new era in CF, full of promise and possibilities. To achieve this potential, effective screening and diagnostic testing must be in place. Prenatal and neonatal screening programs mean that infants can be diagnosed and interventions begun before they are symptomatic. In some countries, CFTR genotyping is frequently performed early in life, and mutation- or class-specific *CFTR* modulators have already changed the lives of older infants and children. What was once unimaginable could become reality—primary prevention of CF might be achievable.

## Author Contributions

AC composed the first draft and did not receive an honorarium or grant to write the manuscript. Both authors listed on the manuscript have reviewed, approved the content of the submission, and take full responsibility for the information provided.

## Conflict of Interest

The authors declare that the research was conducted in the absence of any commercial or financial relationships that could be construed as a potential conflict of interest.

## Abbreviations

cAMP: cyclic adenosine monophosphate
CF: cystic fibrosis
CFTR: cystic fibrosis transmembrane conductance regulator
FEV_1_: forced expiratory volume in one second
IRT: immunoreactive trypsinogen
NBS: newborn screening.
